# Blurred boundaries: sexuality and power in standardised patients’ negotiations of the physical examination

**DOI:** 10.1186/s41077-018-0069-2

**Published:** 2018-06-26

**Authors:** Grainne P. Kearney, Gerard J. Gormley, Diane Wilson, Jennifer L. Johnston

**Affiliations:** 1 Centre for Medical Education, Queen’s University, Belfast, Whitla Medical Building, 97 Lisburn Road, Belfast, BT9 7BL UK; 2 Clinical Skills Education Centre, Queen’s University, Belfast, 2nd Floor Medical Biology Centre, Lisburn Road, Belfast, BT9 7BL UK

**Keywords:** Physical examinations, Power, Patient simulation, Simulated learning environment, Simulated patient, Standardised patient

## Abstract

**Background:**

Working with standardised or simulated patients (SPs) is now commonplace in Simulated Learning Environments. Embracing the fact that they are not a homogenous group, some literature suggests expansion of learning with SPs in health professional education by foregrounding their personal experiences. Intimate examination teaching, whether with or without the help of SPs, is protected by a particular degree of ceremony given the degree of potential vulnerability. However, other examinations may be equally intrusive for example the close proximity of an eye examination or a chest examination in a female patient. In this study, we looked at SPs’ experiences of boundary crossing in any examinations, sensitised by Foucault’s concept of the clinical gaze. We wished to problematise power relations that construct and subject SPs as clinical tools within simulation-based education.

**Methods:**

We collected data from 22 SPs, through five focus groups. Analysis was an iterative process, using thematic analysis. Data collection and reflexive analysis continued iteratively until concepts were fully developed and all theoretical directions explored.

**Results:**

Students and SPs construct simulated teaching consultations by negotiating the unequal distribution of power between them. The SPs themselves discussed how they, perhaps unknowingly, acted in accordance with the discourse of the clinical gaze. However, SPs became disempowered when students deviated from the negotiated terms of consent and they used their agency to resist this. The SPs used strong sexual metaphors to express the subjugation they experienced, as discourses of sexuality and gender played out in the Simulated Learning Environment.

**Conclusion:**

We demonstrate that power dynamics and the clinical gaze can have important consequences within the Simulated Learning Environment. Every physical examination can be potentially ‘intimate’ and can therefore be underpinned by discourses of sexuality and gendered undertones. In partnership with SPs, simulation-based education should create a teaching space that no longer fosters the discourse of the clinical gaze but facilitates students to learn to reflectively navigate, in the moment, the fine line between touching patients versus touching loved ones, and the blurred boundaries that exist in the gulf between sexual contact and benevolent touch.

## Background

The Association of Standardised Patient Educators (ASPE) recently published Standards of Best Practice for those working with human role players in simulation-based education, perhaps more commonly known as standardised or simulated patients (hereafter collectively referred to as SPs) [1]. The role of SPs in medical education commenced in the late 1960s [2], and they now form an integral part of teaching and assessment [3]. Wallace et al. stated that ‘they are not a homogeneous group’ [4] and indeed it is recognised that the terminology used to define them, in addition to their demographics, motivating factors and degree of adoption of professional status vary throughout the world’s health profession schools and according to cultural context.

It has previously been discussed how SPs working within a typical UK institution may sometimes feel dehumanised because some do not see their role as simulated at all, experiencing some or all of the physical and much of the emotional responses that would be expected in a genuine consultation [5]. The same study found that these SP participants held as a cornerstone their sense of vocational identity – of giving something of themselves in order to help medical students and their future patients. For some of the SPs, this is what assuaged the associated discomfort [5]. Other literature shows different motivating factors for SPs; for example, they may feel they benefit from the health knowledge that they gain and from insights that they acquire into the practice of medicine [6], seeing themselves very much as *unreal* patients [7], prioritising the learning needs of the student. Understanding such differing SP views of their work and identity (as ‘faculty proxy’ vs ‘patient proxy’) is a conversation that is continually shifting.

Whilst the position of SPs in the Simulated Learning Environment has evolved in the last 50 years, some literature suggests the need for an even greater shift of thinking away from considering SPs as a teaching ‘technology’, to further embrace their experience and knowledge when educating health professionals [7, 8]. This stands in contrast with a common medical faculty idea of choosing ‘cases’, primarily based on disease process, to teach students [7].

Through tracing the sociocultural history of SPs, we can understand the evolution of their roles through the years in medical education, and more recently in the education of many other health professions. Around the time that Barrows [2] was introducing the idea of SPs as an aid in medical teaching, Stillman was doing some early work with ‘patient instructors’ (real patients) who were trained to teach clinical skills and give feedback to the students [9]. A more modern variation is the involvement of Teaching Associates, at present used in a minority of medical schools [10]. These men and women, who choose to allow students to practise real-life gynaecological and other ‘intimate’ examinations using their own bodies, are trained to teach the students and provide feedback in a supportive environment [11]. Previous work done on the experience of patients using their bodies to teach pelvic examination found that taking part had benefits in terms of *a strong sense of self* [12]. Improvements in students’ clinical skills and confidence have been separately shown from such a programme [13]. In an interview carried out by Brian Hodges, Danny Klass, then Associate Dean of the University of Manitoba, acknowledged the sensitivities surrounding teaching such examinations. In describing the adoption of their real-patient gynaecological teaching programme, it was his opinion that these women ‘sacrificed themselves for this teaching’ [14].

SP’s experiences and personal agency come sharply into focus in teaching and assessing intimate examinations, such as breast, pelvic or rectal examinations. Indeed, the particularities for SPs involved in this simulated teaching are highlighted by the fact that this is one of a few specific areas that the ASPE intends to publish further specific standards in [1]. Because of the nature of these examinations, which have increased potential to be open to misinterpretation, they are often taught with particular emphasis on protecting both patient and doctor. Such an examination performed without appropriate consent can be misconstrued and even cross into the realms of sexual assault. Furthermore, terminology is thus kept very technical and references to sexuality are minimal. To avoid SP embarrassment, hybrid simulation, typically involving a plastic mannequin for example of a rectum, is often used in conjunction with an SP [15]. Students are taught a specific, semi-formal ritual around intimate examinations, including the role of chaperones, alongside the technical skill itself [16].

Following some high-profile cases where practising doctors’ clinical approaches were misconstrued as sexual advances [17], we became interested in exploring the teaching of examinations which may traditionally be considered less ‘intimate’ by doctors, teachers or patients, but may still potentially cross the personal boundaries of the patient. Guidance published by UK regulatory body, the General Medical Council (GMC), recognises this potential as inherent in any examination, including those not typically considered intimate: ‘whenever you examine a patient you should be sensitive to what they may think of as intimate. This is likely to include examinations of breasts, genitalia and rectum, but could also include any examination where it is necessary to touch or even be close to the patient’ [16]. The patient may thus still be exposed to a potentially significant degree of embarrassment and distress in a range of ‘ordinary’ examinations [16]. Examples might include exposing a woman’s chest for respiratory examination, examining femoral pulses or the enforced proximity of an eye examination using an ophthalmoscope in a darkened room. Furthermore, it is likely that such examinations may be especially poignant for patients who have experience of trauma; clinical assessment by well-intended health care providers risk re-traumatising such individuals. Whilst there has been much coverage of these alleged sexual assault cases through the popular media, this has not been significantly reflected in research or the academic medical literature.

In this study, we looked at SPs’ experiences of examinations which involved a substantial invasion of personal space and explored issues of power. We were interested in whether there is recognition of the degree of boundary crossing (touch into the personal space of the patient, as defined by the patient) in each and every examination regardless of whether it is labelled ‘intimate’ or not. Our analysis was sensitised by Foucault’s concept of the *clinical gaze* [18, 19]. This describes the modern work of medical practice as a discourse where the doctor is powerful as a result of their scientific knowledge and training. Focusing on the ‘technical’ aspects of clinical care, the patient’s physical self is considered separate from their mental and spiritual selves. In the discourse of the clinical gaze, patients may be dehumanised, with their stories and embodied experiences disregarded in favour of objectivity and technical expertise [20]. Martimianakis and McNaughton state that ‘The application of the medical gaze is a form of boundary work that is intimately linked to medicine’s professionalization project’ [21]. Hodges argued that modern medical education had indeed redirected the medical (clinical) gaze onto the body of the student, calling it the ‘inevitable extension of the medical gaze into the classroom’ [22]. He stated that the physician/teachers work to ‘know students bodies’ [22] which is ‘to understand the mechanics of learning and being a trainee in order to be able to diagnose diseases of the curriculum’ [21]. We were interested in how SPs perceived the relationships between their ‘bodies’ and medical students in the Simulated Learning Environment.

## Methods

This study was approved by the School of Medicine, Dentistry and Biomedical Sciences Research Ethics Committee in Queen’s University, Belfast (ref 13.03v2).

### Setting

The setting was the undergraduate medical degree programme at Queen’s University, Belfast where just over 100 SPs are registered for teaching and assessment in simulation based education throughout the 5-year program. In the mainly ‘pre-clinical’ years 1–2, medical students encounter SPs weekly in teaching and in their end of year assessments. In the ‘clinical’ years, the majority of encounters with SPs are in the context of assessment only. The SPs enter into a contract with the University and are paid for their time.

### Research team and reflexivity

GK is a General Practitioner and clinical teacher and was a postgraduate student at the time. GG, DW and JJ are clinical academic General Practitioners. The research team came together due to a common interest in teaching with SPs. Some of the team have particular expertise in simulation including SP methodology and have published in this area previously. (GG, JJ) The research team, whilst known to the SP group, were not involved directly in their training or management. The team maintained high levels of reflexivity (checks) during data analysis by conducting regular critical discussions and reflecting on their own subject positions relative to the research.

### Recruitment and sampling

SPs who were actively involved in clinical skills teaching for second year students during the period of data collection were all invited by email to participate in the study and the focus groups took place at a time and location convenient to their teaching commitments. It is important to note that these SPs are involved with students in all 5 years of the course and no emphasis was placed on the particular teaching session or the students that they were due to teach on that day during the discussions. We involved all SPs (*n* = 22) who expressed an interest in taking part and did not recruit further after analysis as we felt that saturation had been reached. These SPs represented the range of age, gender and experience of the whole SP cohort in our institution (Table 1). Participants gave informed, written consent.

**Table 1:** Participant’s demographic characteristics

Demographic characteristic	Participants* (*n*)
Gender	
• Male	13
• Female	9
Age (years)	
• 41–50	2
• 51–60	6
• > 61	14
Experience as a SP (years)	
• < 1	3
• 2–3	6
• 4–5	6
• > 5	7

*Total *n* = 22

### Data collection and analysis

Data was collected by GK and JJ between April and June 2013, through five mixed gender focus groups, containing between two and six participants, giving 197 min of interview data in total. The topic guide was discussed and agreed within the research team prior to the focus groups, it then developed iteratively throughout the process. The topic guide was based on participants’ experiences of clinical examination, with particular attention to boundary-crossing in examinations. The questions were purposely broad to try to prevent only negative experiences being described and to reduce any ‘competitiveness’ in the focus groups. The potential for discussions to involve sensitive topics was recognised; however, SPs were well briefed about the nature of the research in advance including an emailed participant information leaflet and at the start of the focus group and appeared comfortable within a small group of their peers. It was also felt that the supportive co-construction of the group seems to encourage SP narratives and contributed to their engagement. SPs discussed examinations that they had taken part in as part of their teaching roles (they are generally not involved in intimate examinations in this institution with the exception of some female SPs who chose to be involved in breast examination teaching). Throughout the focus groups, the SPs chose particularly to refer to their teaching experiences in chest examinations (in female patients), breast examinations, abdominal examinations and examinations of the femoral pulse. They also chose to draw at times on their experiences as patients being examined in intimate examinations and otherwise, as had been the case in previous research carried out with SPs [5]. Contemporaneous field notes were made. Interviews were audio-recorded, anonymised, transcribed verbatim and checked for accuracy by GK. Quotations, below, are coded: FG(n) *[Focus group number]*, F or M *[female or male participant].*

We undertook a thematic analysis [23], conducting analysis concurrently with data collection. Analysis involved coding and memo-writing, with data collection and inductive analysis continuing until saturation. GK carried out initial coding. The research team met regularly to advance the analysis until all concepts were well developed, and all relevant theoretical directions had been explored. Theoretical links were only explored after the bulk of inductive analytic work was complete as suggested by the data and are recounted below.

## Results

Analysis yielded three main themes: boundary negotiation, boundary violations and protecting boundaries.

### Boundary negotiation

SPs communicated their experiences of boundary crossing as a negotiation of power and consent between the SP and the medical student. SPs described an expectation that students would actively modulate their actions and respond to SP feedback. This was considered necessary for SPs to permit students to proceed with examination, regardless of whether this was a routine or intimate encounter. The research highlighted a need to navigate sexuality in the physical proximity of these boundary crossing examinations (though there was also some discussion of examinations where they felt that sexuality was not a major issue). Students were often perceived by the SPs as experiencing a particular level of embarrassment or discomfiture in examinations which encroached on ‘taboo’ body areas, even if these were not classed as intimate examinations.

‘And especially if they’re taking just below the waistband [taking femoral pulses] – some don’t want to do that. You know they don’t want to do that, and they might try and take the pulse through the shorts.’ (FG5, M)

SPs recognised that these examinations, involving breaking personal space, were frequently difficult for students in this Simulation Learning Environment. Affording students a safe space in which to practise negotiation of boundary crossing with the SPs manifested as a central SP role. Indeed, SPs described instances where they subjected themselves as teaching tools in order to facilitate students’ learning; in essence, the SPs themselves were acting in accordance with the discourse of the clinical gaze.

*‘*There is your patient semi-naked – what part of that patient do you not like to touch? Well he’s quite happy so touch it now! Get so used to it that it’s not a problem to you.’ (FG5, M)

SPs tried to bridge the experience gap between themselves and the students, who they perceived as being young and inexperienced. They brought their embodied, personified life experience to bear on teaching. The nature of some situations that they described and some experiences that they recalled suggested that they were using their ‘life experience’ actually as a euphemism for sexual experience, and that they were implicitly contrasting this to the ‘life experiences’ of the students that they encountered.

‘At my age, lots of things have been done in real life!’ (FG4, M)

‘So, whenever someone who has come from, shall we say, a sheltered background is presented with some guy or girl lying on a bed with a bra and pants or whatever – how do you deal with that? Because you’ve never experienced it before.’ (FG5, M)

SPs described occasions where they felt that students’ preoccupation with avoiding any possible inference of sexual misconduct could lead to them neglecting routine tasks of the consultation, such as negotiation of power, interfering with the forming of a clinical relationship between the two.

### Boundary violations

SP consent was predicated on the process of negotiated boundary crossing during their interaction with the student, within predetermined limits, such as what had been discussed at training or what previous students had requested. Whilst the SPs did describe how some students, through fear of their actions being misconstrued, neglected to perform a full examination, they also frequently spoke about times where there was seemingly little recognition of their personal space. Personal boundary invasions happened flippantly in these cases. Pushed beyond these limits, into violations of their boundaries, the SPs became acutely uncomfortable. SPs used particular sexual metaphors to describe their experience of being examined by students, drawing emphasis to their perceived passivity and lack of agency.

‘As you say, some of them pull back the curtains and then you’re left there like a piece of meat at the end of it.’ (FG 4, F)

Dysfunctional encounters were most likely to occur with a female SP and male student, though there were a small number of narratives within the same gender or with a female doctor and male SP. There was some indication that tension within these interactions was interpreted slightly differently between the genders of SPs.

‘With a male, there is...maybe the sexual thing, the intimidation thing.’ (FG2, F)

Any deviation from the initial boundary negotiation, even if perceived as minor such as an extra student attending, changed the terms of consent and had the potential to lead to intense feelings of dehumanisation and loss of agency regardless of gender of the student or SP. SPs felt that they should be in a pivotal place in a consultation and felt disempowered when this appeared not to be the case.

‘You’re immaterial, we’re only here to examine you but will not tell you what the findings are, you’re not here at all. So, you’re nobody, but I should be the centre of attention.’ (FG 1, M)

We observed that the SPs attempted to interrupt the subjectification that was occurring through the clinical gaze, by asserting their individuality and identity.

### Protecting boundaries

Once within the teaching session, SPs were often positioned within a relatively passive role, often with a predefined script, enhancing their vulnerability. At times, SPs worried about maintaining control during the teaching session, and attempted to address this by preparing ahead of time. This included having their voices heard in preliminary training sessions, where they offer opinions about the specifics of the examinations involved and wearing appropriate clothing. In this circumstance, their clothing felt protective, and disrobing became a symbolic action.

‘The good thing about that – there was very good training… part of the discussion at the training [was] about the kind of cape we would wear Which I thought was very good’ (FG3, F)

SPs used clothing as a means of asserting their own agency, for example by covering up if they felt that students had asked for an unreasonable amount of exposure.

‘… and I’ll say ‘no it’ll do to here’ [regarding exposure of the abdomen].’ (FG4, F)

SPs felt that if they had been properly prepared for and consulted about the encounter in advance, then the consultation generally went well. However, if insufficient attention was paid to these steps, they felt exposed, disempowered and defensive.

‘As you say, only uncover the bit that needs examined! ... And then they say ‘Oh right, pull that back down again’ and then you feel okay. So as long as you’re only exposing the bit that needs examined, I think you can sort of cope with that bit.’ (FG4, F)

SPs’ embodied experience, their personal expression of the embarrassment and discomfort of examination contrasted sharply with traditional teaching of examinations where the person is separate from the body. Below, this SP quotes a student using a ‘textbook’ description of abdominal examination [24]:

‘[student] “Can you take everything off, ideally nipples to knee?”’ (FG4, F)

In this instance, we see the discourse of the clinical gaze active in the student’s choice of language, echoing the medical textbook jargon ‘from nipples to knees’. This language subjects the patient as a clinical object or collection of body parts rather than as a whole person, and neglects the extreme embarrassment felt by exposing more than is necessary. When challenged in the way, SPs reasserted their agency by any means available to them. This included deeming the student’s request as unreasonable and refusing to do as asked, sometimes whilst looking to the tutor in the session to ensure that this request was retracted.

## Discussion

### Power in the physical examination

In this study, SPs construct boundary negotiation in teaching examinations as a crucial process in simulation based education. SPs are at risk of objectification, even at their own hands, and they described examples in this study of how they actively chose to subordinate themselves in order to facilitate learning. However, SPs’ embodied experiences may challenge assumptions that they can be viewed as malleable, willing tools with which to complete teaching work [2, 7] as they try to work as active agents to maintain a sense of control within the simulated consultations. Evidence has shown that many SPs strive to be considered active teachers, rather than passive technology, [7, 8] so why do SPs at times still appear to subject themselves, under the clinical gaze, to subordination? What are the unspoken power dynamics promoted in simulation based education which enables this to happen?

### Power and sexuality

The medical community often accepts that examinations of breasts, prostates and genitalia, constitute an invasion of personal space [16]. The intimacy attached to such examinations is defined culturally by discourses of sexual behaviour which render these body parts ‘taboo’, whilst others are not. In this research, however, SP experiences beyond the classic ‘intimate’ examinations were heavily influenced by discourses of gender and sexuality; we refer to sexuality as the capacity for sexual feelings, not as sexual orientation. SPs of both sexes (though predominately the female SPs) described instances of boundary violation in simulated examinations, where boundary negotiation had not been successful within the clinical encounter. Such dysfunctional encounters with students were on occasions narrated using evocative sexual metaphors. These are more commonly used within Western culture to describe a subordinate female position within a sexual relationship [25]. Drawing on a discourse of female sexuality, [26, 27], SPs of both sexes, in using this language appeared to position themselves in an inferior position as a way of expressing their loss of power and their subjugation. Are faculty deliberately avoiding these sensitive discussions with teachers, students and SPs? Whilst discourses of sexuality, gender and subjectification are not likely to be unique to Simulated Learning Environments, could these important areas be considered during session planning and opportunities to help address them created in an attempt to highlight issues that may be present throughout teaching and learning?

### Control and agency

Navigating unspoken sexual undertones became a central task for SPs in their experiences of examinations through their narratives. This often played out in a power tussle between student and SP surrounding the degree of necessary exposure for a particular examination. Significant tension was evident between the textbook exhortation for the student to ‘expose the patient nipple to knees’ [24] for abdominal examination. When the boundaries of the SPs were challenged, they displayed their discomfort by asserting their individuality and agency as human beings through deliberate non-compliance with the instructions dictated by the student. In addition, clothing became an important representation of their agency, and asserting control over disrobing and its extent became a symbol of power struggle between the parties. They described how they resisted the classic medical formulation of bodies as separate entities from their occupants, and illness as purely mechanical dysfunction [18, 28], through their personal narratives expression and desire to be at the centre of their care.

### Implications for medical pedagogy

Simulated Learning Environments have the potential to profoundly influence students’ learning about boundaries and power relations towards future clinical encounters, but it must be acknowledged that even in simulated consultations, there is debate about the relative power held by students [29, 30]. In some instances, by definition of their ‘standardised’ roles, SPs may still follow scripts and behave in a manner which is circumscribed by doctors [2]. McNaughton argues that this stands in direct opposition to the normal behaviour of patients in clinical relationships which, whilst culturally defined, is not usually controlled by the clinician to such an extent as some SPs may be at times when ‘trained’ for their profession [8]. It is important to recognise that this has the potential to control and limit the SP’s space for agency.

Given that issues of power and sexuality are so embedded in examination and its teaching, it is interesting to note that the history of standardised patients is firmly rooted in discourses of gender and sexuality. In fact, commercial sex workers were precursors to today’s SPs, often employed as gynaecology demonstrators in medical schools up to and during the 1970s [31]. This practice, which may seem so unacceptable to modern sensibilities with its sterile pedagogy, underlines the idea that embodied experience may be bought and objectified for medical gain. Simulated teaching consultations may easily serve to reify power inequalities and transfer them to a new generation of health care professional. This is a heavy responsibility for health profession education to bear, which could be addressed by greater inclusion of patient voices in education. Just as we are moving towards shared decision-making and exchange of expertise in real-life consultations, so too should we in simulated educational contexts. Effort is required to promote a simulated teaching environment where SPs are reassured that their work is alongside the clinical educators, where they do not feel the need to construct themselves as pedagogical tools. Using real demonstrators, rather than relying on hybrid simulation alone, denotes a commitment to the non-technical, communicative aspects of clinical skills in simulation based teaching and reaffirms the patient’s role as active partner in the co-construction of the consultation. In developing these roles, SPs should be afforded training and support accordingly, with full acknowledgement of their agency and by encouraging them to halt any examination (or any other simulated activity) if they feel that they are being dehumanised. This role development could be achieved through increased level of SP involvement throughout curricula planning and through awareness of their emotional commitment. Educators must be prepared to attend to the less sterile and rehearsed aspects of these examinations with students, working with the SPs in a simulated teaching context. The effort must include education for both the teachers and the students involved in these Simulated Learning Environments, to ensure that the Standard of Best Practice are being implemented both in spirit and in practical terms [1].

More prosaically, students need opportunities to learn the fine line between how they touch a patient compared to the touch of a loved one; in addition to the crucial gulf between a therapeutic touch and a violation, as described by the SPs in this study. Countering such powerful and entrenched forces requires revolutionary change in the way in which we view patienthood. The separation of consultation skills from content knowledge is common and unhelpful [32]. Instead, we should teach them as inevitably entwined in the construction of clinical encounters. Examination is a fundamental means of communication, and an important type of social work undertaken within the consultation.

Lastly, and most profoundly, we build on these results to assert that any examination experience should be treated as a potentially ‘intimate’ invasion of the person in front of them. Indeed, the principal of ‘respect’ is specifically emphasised under one of the domains of the Standards of Best Practice; to ‘Respect SPs’ self-identified boundaries (e.g. modesty, limits to physical touch, impact on person)’ [1]. Students should learn to address examination appropriately in terms of judging and negotiating appropriate boundary crossing, with the basic understanding that all examinations may fall at different places in the continuum from no intimacy to fully intimate dependent on the context. Consent is predicated on informed agreement between agent parties; students must learn that this point of intersection is not static, but a dynamic process which continues throughout the consultation.

### Strengths and limitations

We situate our research within the constructionist paradigm, and so whilst we have aimed for transferability to similar contexts, we do not intend to generalise. As with any qualitative research, the findings are highly situated, in this case to their UK context. We recognise that the demographics and characteristics of participants here are not necessarily representative of other educational settings, particularly those of North America where SPs may occupy a qualitatively different role.

All members of the research team are clinical teachers and would have been known to participants in this guise. It is certainly possible that traces of a patient-doctor power gradient transferred to the researcher-participant relationship, and this is likely to be inherent in the relationship rather than related to researchers’ own styles of questioning. In performing analysis, all researchers were cognisant of their various roles, experiences and potential biases, and reflected on them through a research diary and in meetings throughout the process. A form of thematic analysis was used, within an iterative research process. We recognise the central role that an emphasis on reflexivity must play in this study but feel that the situatedness of researchers is considered overall to be a strength of the research. In addition, whilst the method of focus groups was chosen to encourage supportive discussion within a group of peers, the risk of this resulting in competitive narratives was considered a possible consequence. Nevertheless, we did find that the SPs were keen to share their rich personal narratives of experience.

Much more research is needed into the important social and cultural nuances of working with SPs, and of the ways in which we teach the consultation to medical students. Future theoretical perspectives may include formal discourse analysis, a specific orientation to feminist theory, or simply a phenomenological exploration of SP experience. Additionally, the perspectives of other stakeholders, such as students and teachers in such examinations would be worthwhile exploring.

Most importantly, we did not have an SP as part of the research team and have learned from our own results that this should be a priority for future research projects. Stakeholder involvement in research is commonplace in clinical research but not yet in medical education. This is something which we hope will not just influence our own work, but also that of others in the field.

## Conclusion

There is as yet little recognition within simulation based health profession education of the influence of gender, sexuality and power in the most mundane of clinical interactions. All examinations are imbued with power dynamics, and the necessary proximity of human bodies means that this often takes on a sexual or gendered undertone in terms of any examination. Educators should encourage understanding that any examination could be considered intimate and so consider how to offer students a deeper and more meaningful experience in the Simulated Learning Environment. Working in partnership with Simulated Patients can offer challenge to the discourse of the clinical gaze and help students learn to thoughtfully negotiate the blurred boundaries that exist between sexual contact and benevolent touch.

## Abbreviations

ASPE: The Association of Standardised Patient Educators
FG: Focus group
GMC: General Medical Council
SP: Standardised/simulated patient
